# Reducing the effects of aging on cognition with therapeutic intervention of an oral multi‐nutrient combination (REACTION): A pilot study – Report of primary feasibility outcomes and quality of unblinding

**DOI:** 10.1002/alz.093287

**Published:** 2025-01-03

**Authors:** Christian J Camargo, Katalina F McInerney, Stacy S Merritt, Marisa Modjeski, Tatjana Rundek

**Affiliations:** ^1^ University of Miami Miller School of Medicine, Miami, FL USA

## Abstract

**Background:**

Age‐related cognitive decline (ARCD) refers to the cognitive changes that occur in individuals because of aging. Research suggests that the underlying mechanism behind ARCD is a loss of synaptic plasticity and altered dendritic spine morphology. Similarly, the cognitive changes in Alzheimer’s Disease (AD) are also thought to arise from impaired synaptic plasticity and dendritic spine loss. A specific combination of precursors and cofactors for phospholipid biosynthesis has shown beneficial effects on memory, cognition, and brain atrophy in prodromal AD. To test the feasibility of this multi‐nutrient combination intervention in ARCD, the REACTION pilot study was developed.

**Method:**

REACTION is a 6‐month single‐center randomized, double‐blind, placebo‐controlled parallel group virtual pilot clinical trial. The study factors in local COVID‐19 restrictions and trial visits are done virtually using secure online video communication. Eligible subjects are English or Spanish speaking people aged 55−89 years from all ethnic groups with ARCD and screened and randomized to the oral multi‐nutrient combination (Souvenaid) or placebo on a 1:1 basis. The main outcome is feasibility (recruitment rate, recruitment time, adherence rate and retention rate). Exploratory outcomes include memory (e.g., Ray Verbal Learning Task), performance on other cognitive tasks (e.g., Oral Trail Making Test), and quality of life (e.g., Amsterdam IADL Questionnaire) outcome measures.

**Result:**

In JPAD (Camargo et al., JPAD. 2023;10:821‐7), we published our final pre‐specified criteria for feasibility. We report that we met pre‐specified criteria for “feasible” for recruitment rate, recruitment time, and study retention. We are unable to draw conclusive data on adherence to intervention due to staff shortages for data acquisition. After unblinding, for the placebo‐controlled portion of the study, we report that our randomization was successful, e.g., with matched age (69.8 years ± 9.12 [STD] and 68.5 years ± 8.67 [STD], p = N.S.), years of education (17.3 ± 2.38 [STD] and 16.69 ± 2.36 [STD], p = N.S.) for placebo and active groups, respectively. This will enable exploratory analysis of efficacy outcomes of cognitive and behavioral data.

**Conclusion:**

This study shows that conducting a fully virtual study with a nutritional intervention to test its effect on ARCD progression is feasible in persons with ARCD.

